# Neurogenesis in the olfactory bulb induced by paced mating in the female rat is opioid dependent

**DOI:** 10.1371/journal.pone.0186335

**Published:** 2017-11-06

**Authors:** Marianela Santoyo-Zedillo, Wendy Portillo, Raúl G. Paredes

**Affiliations:** Instituto de Neurobiología, Universidad Nacional Autónoma de México Campus Juriquilla, Querétaro, México; Duke University, UNITED STATES

## Abstract

The possibility to control the rate of sexual stimulation that the female rat receives during a mating encounter (pacing) increases the number of newborn neurons that reach the granular layer of the accessory olfactory bulb (AOB). If females mate repeatedly, the increase in the number of neurons is observed in other regions of the AOB and in the main olfactory bulb (MOB). It has also been shown that paced mating induces a reward state mediated by opioids. There is also evidence that opioids modulate neurogenesis. In the present study, we evaluated whether the opioid receptor antagonist naloxone (NX) could reduce the increase in neurogenesis in the AOB induced by paced mating. Ovariectomized female rats were randomly divided in 5 different groups: 1) Control (not mated) treated with saline, 2) control (not mated) treated with naloxone, 3) females that mated without controlling the sexual interaction (no-pacing), 4) females injected with saline before pacing the sexual interaction and 5) females injected with NX before a paced mating session. We found, as previously described, that paced mating induced a higher number of new cells in the granular layer of the AOB. The administration of NX before paced mating, blocked the increase in the number of newborn cells and prevented these cells from differentiating into neurons. These data suggest that opioid peptides play a fundamental role in the neurogenesis induced by paced mating in female rats.

## Introduction

The ability that female rats have to control (pace) the rate of sexual stimulation has been observed in natural, semi-natural and laboratory conditions [1–5]. There are clear physiological and behavioral advantages when females pace the sexual interaction. For example, they show higher levels of prolactin release after mating, have higher pregnancy rates and sire more pups than females not pacing the sexual interaction [1, 6]. It has also been demonstrated that when females [5, 7] and males [8] paced the sexual interaction they developed a positive affective, reward, state as evaluated by the conditioned place preference paradigm (CPP). When the opioid antagonist naloxone was administered before females [9, 10] or males [11] paced the sexual interaction they did not develop CPP, suggesting that the reward state induced by paced mating is mediated by opioids.

Another physiological consequence associated with paced mating is that it induces neurogenesis. Sexual behavior in male rats induces a higher number of new cells in the dentate gyrus of the hippocampus [12] and in the granular layer of the accessory olfactory bulb [AOB; [13]]. Interestingly, the increase in the number of cells and neurons observed in the AOB either 15 [13] or 45 [14] days after mating was observed only when the males controlled the rate of the sexual interaction. An increase in neurogenesis is also observed in females when they pace the sexual interaction. One paced mating encounter induces a higher number of cells that differentiate into neurons in the granular layer of the AOB [15]. If the stimulus is repeated and the females mate 4 times in a 16-day period, a higher number of cells and neurons is observed in the granular and mitral layers of the AOB and in the granular layer of the MOB [16]. Together, these results indicate that the ability to control the rate of sexual interactions in males and females is crucial for the induction of neurogenesis in the OB.

Adult neurogenesis has been studied and documented throughout the life span of mammals. The most studied regions incorporating new neurons in the adult brain are the dentate gyrus of the hippocampus and the OB. In the case of those that reach the OB, neuronal progenitor cells are located in the postnatal subventricular zone (SVZ) of the lateral ventricles; they proliferate, migrate and incorporate as interneurons in the granular layer or glomerular layers [17–19]. Stem cells and progenitors are regulated by intrinsic factors that control proliferation rates and the fate of newborn cells. One of the factors that regulates the process of neurogenesis in the hippocampus and the SVZ-OB system is opioids. An injection of morphine increased the incorporation of the DNA synthesis marker 3H-thymidine into the DNA of the rat striatum an effect that was blocked by the opioid antagonist naloxone [NX] [20]. Studies in vitro have also shown that morphine induces neuronal and glial differentiation, effects that were blocked by NX [21]. In addition, NX and other mu and delta opioid antagonists blocked 3H-thymidine incorporation in in-vitro cultured rat hippocampal progenitors [22]. In the present study, we evaluated if the administration of the opioid antagonist NX, in a dose that blocks the rewarding state induced by paced mating, can also block the neurogenesis induced in the AOB after the first session of paced mating. This would allow us to determine whether opioids modulate the neurogenesis process in a natural occurring behavior that is fundamental for the survival of the species.

## Material and methods

### Animals

Fifty sexually naive female rats (Wistar) weighing 200–230 g from a local colony, (originally acquired from Charles River) were used. They were housed in groups of 3 or 4 rats per cage. All animals were maintained under a reversed 12-h light/dark cycle with food and water ad libitum. They were bilaterally ovariectomized, two weeks before the experiments, under general anesthesia using a mixture of ketamine 70% and xylazine 30% (1 ml/kg per rat). To induce sexual receptivity, female subjects received a subcutaneous injection of estradiol benzoate (25 mg/rat–Sigma-Aldrich) and progesterone (1 mg/rat–Sigma-Aldrich), both diluted in corn oil, 48 h and 4 h, respectively, before each behavioral test. Experimental females were randomly distributed in five experimental groups (see below). Sexually experienced male rats (Wistar) weighing 300–320 g were used as stimulus in sexual behavior tests. They were trained using other sexually receptive females not used in this experiment.

All experiments were carried out in accordance with the ‘‘Reglamento de la Ley General de Salud en Materia de Investigación para la Salud” of the Mexican Health Ministry, which follows NIH guidelines and were approved by the Bioethics Committee of the Instituto de Neurobiología (INEU/SA/CB/040).

### Behavioral tests

The behavioral tests were done in Plexiglass chambers (40 cm X 60 cm X 40 cm). Subjects, bilaterally ovariectomized and hormonally supplemented, were randomly assigned to one of five groups: 1) Control saline, females without sexual contact injected with saline; 2) Control NX, females without sexual contact injected with NX; 3) No pacing, females injected with saline that mated without pacing the sexual interaction; 4) Pacing saline, females injected with saline before paced mating and 5) Pacing NX, females injected with NX before paced mating. The control groups did not receive any sexual stimulation, and were placed alone in the testing chamber for 60 min (groups 1 and 2). In groups 4 and 5, females mated with a sexually experienced male in a pacing chamber that allowed the female to control (pace) the sexual interaction. In this case, the male and female were separated in the test chamber by a removable plexiglass partition with a small hole (7 cm in diameter) near the bottom that allowed the female to freely move back and forth from the male side. The hole was too small for the male to go through. In the non-paced group the animals mated freely without the plexiglass partition. In this case the male, not the female, controlled the rate of the sexual interaction. All tests lasted 1 hour. After the test, females were returned to their home cages for the next 15 days with other females (3 or 4 females per cage) of the same group.

### Naloxone injection

The opioid antagonist NX hydrochloride (Du Pont, México) was injected i.p. in a volume of 1 ml/kg of NaCl 0.9%. The compound was injected in a dose of 4 mg/kg 1 min before females paced the sexual interaction. This dose blocked the reward state induced by paced mating without affecting sexual behavior [10].

### BrdU injections

The day of the behavioral tests, all females received 300 mg/kg of the DNA synthesis marker 5’bromo2’deoxyuridine (BrdU, Sigma) dissolved in NaCl 0.9%. They were injected i.p. three times, 100 mg/kg per injection, as follows: 1) 60 minutes before the test, 2) immediately after testing and 3) 60 minutes after the test.

### Tissue preparation

The animals were sacrificed, with an overdose of anesthetic, 15 days after the test and intracardially perfused with 0.1M phosphate-buffered solution (PBS, pH 7.2) followed by 4% paraformaldehyde (Sigma-Aldrich). The brains were collected with special care to avoid damage to the olfactory bulbs, then post fixed for 60 min in paraformaldehyde 4% and transferred to 30% sucrose in PBS 0.1 M until histological processing. To detect BrdU-positive cells in the MOB and AOB, floating immunostaining was carried out on 30 μm thick sagittal brain sections obtained with a Leica microtome.

### Immunohistochemistry

We followed a previously described protocol [13, 15, 16]. Briefly, the sagittal sections were washed 4 times in Tris-buffered saline (TBS 0.1 M, pH 7.6 J.T Baker) and incubated in sodium borohydride (0.5%, Sigma-Aldrich). They were then rinsed in TBS and incubated for 30 min in Triton X-100 (0.1%, J.T Baker) and 30% H_2_O_2_ (1%, J.T Baker). Later, sections were incubated in 1% dimethyl sulfoxide for 10 min (DMSO, J.T Baker). Tissue was rinsed in TBS and incubated in 2 N HCL (J.T Baker) at 37°C for 60 min and incubated again in sodium borohydride for 15 min. To block the unspecific epitopes, sections were incubated for 30 min in 0.1% bovine albumin and 0.03% Triton X-100. The tissue was incubated for at least 16 h at 4°C in primary antibody, mouse monoclonal anti-BrdU (1:2000, BD Biosciences) in 0.1% bovine albumin and Triton X-100 (0.03%). The sections were then rinsed with TBS and incubated with secondary antibody: biotinylated anti-mouse IgG (1:500, Vector Laboratories,) diluted in TBS with Triton X-100 (0.032%) and bovine albumin (0.1% for 2h at room temperature). The tissue was then incubated in Avidin Biotin Complex (ABC elite kit, Vector Laboratories,) for 90 min. Brain sections were rinsed and incubated with nickel chloride-3,3'-diaminobenzidine (DAB, Vector Laboratories) and H2O2. The reaction was stopped by washing the sections in TBS. Finally, the brain sections were mounted on gelatin-coated slides and cover slipped using permount (Fisher Scientific).

### Quantitative analysis of DAB staining

BrdU-DAB stained tissue images (10X objective) were obtained with a light microscope OLYMPUS BX60F-3 connected to a motorized slide (Prior ProScan) and analyzed using Image Pro Plus 6.1 software. BrdU-IR cells were identified and quantified in every third section of the MOB and AOB. The glomerular, mitral, and granular layers were analyzed. We quantified the anterior and posterior regions of the granular layer of the AOB because there is a clear anatomical, functional and chemical distinction between them [23, 24]. The area of interest (AOI) in both the MOB and AOB was delimited with three circles in each layer. The diameter of the circles used in the MOB and AOB was 400 μm and 200 μm respectively. The total AOI per layer was calculated with the sum of areas in each circle. The number of new cells was obtained with the average number of BrdU-IR positive cells in the total AOI per layer. We quantified four sections per brain (n = 7 subjects per group).

### Phenotypic identification of the new cells

**Fig 1:**
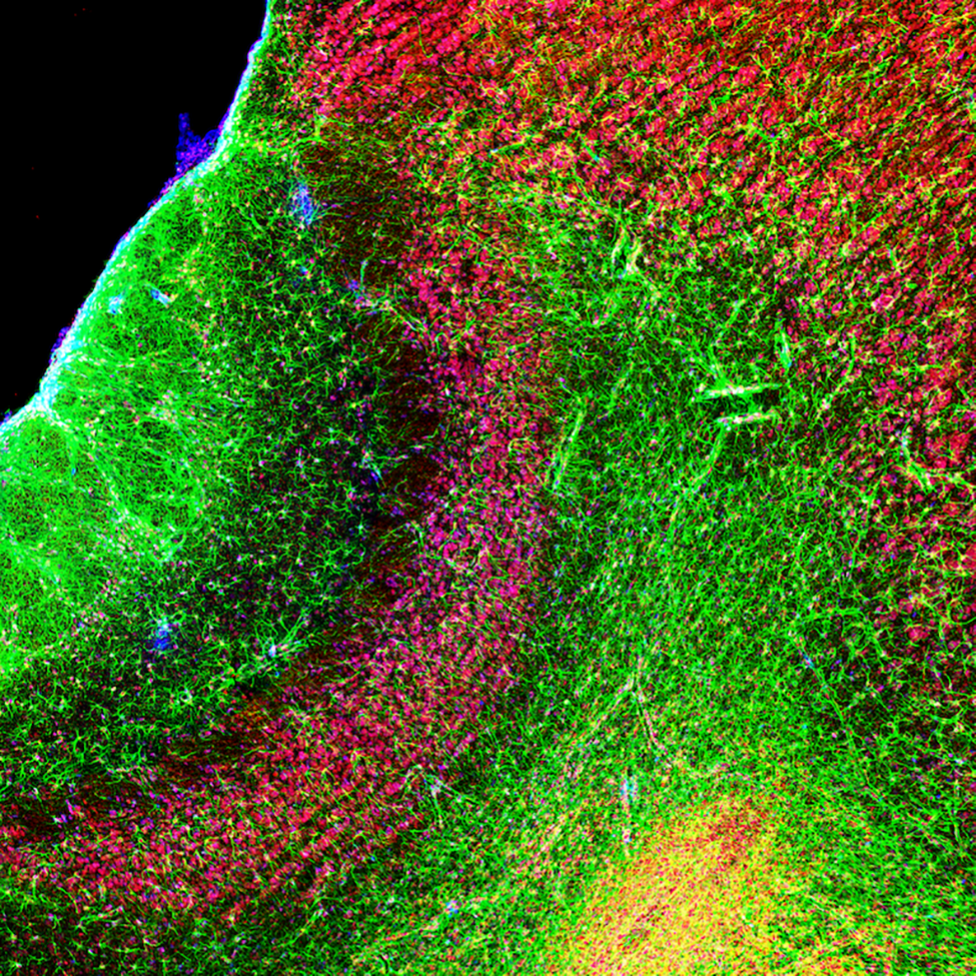
Triple label immunoreactivity in the accessory olfactory bulb. Sagittal section of the AOB (20 x) in a female rat showing immunoreactive cells for GFAP (green), NeuN (red) and BrdU (blue).

Triple staining was performed to identify the phenotype of the new cells (Fig 1).

Sections were processed following the immunohistochemistry protocol described above. Samples were incubated in primary antibody rat anti BrdU (1:800, AbD Serotec) for at least16 h at 4°C followed by incubation for 2 h with the secondary antibody rat IgG (1:500, Vector). After incubation, the samples were placed in ABC and revealed with tyramide signal amplification (TSA) plus coumarin system (1:100, PerkinElmer) according to vendor’s instructions. Sections were rinsed and incubated in mouse monoclonal anti- neuronal nuclei antibody (NeuN) to identify mature neurons (1:250, Millipore) for 20 h at 4°C and incubated in mouse IgG (1:300, Vector). Later, samples were incubated in ABC complex and revealed using TSA plus cyanine 3 (CY3) system (1:100). Sections were rinsed and incubated with primary antibody rabbit monoclonal anti- glial fibrillary acidic protein to identify glial cells (GFAP, 1:100, AbD Serotec) and incubated with secondary anti-rabbit IgG Alexa Fluor 488 (Life science, 1:1250). Finally, brain sections were mounted on slides and cover slipped using aqua poly/mount (Polysciences).

### Quantitative analysis of immunofluorescent staining

**Fig 2:**
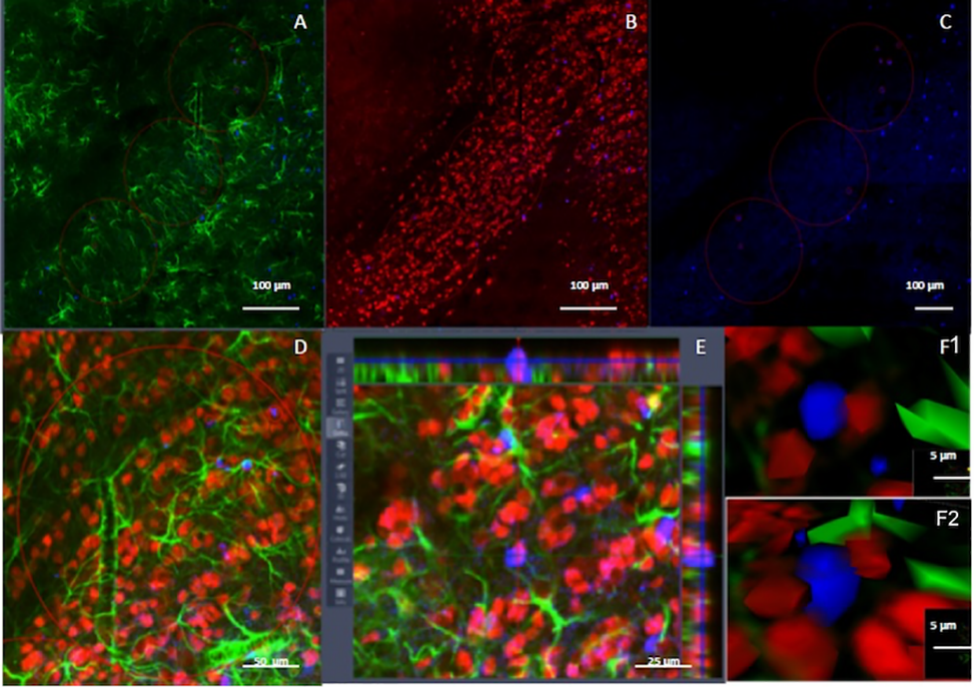
Confocal analysis of the phenotype of the new cells in the granular layer of the accessory olfactory bulb. Acquisition of confocal images in the granular layer of the AOB including immunoreactive cells for GFAP (A, green), NeuN (B, red) and BrdU (C, blue) including a higher magnification (D), an orthogonal view (E) and a 3-D reconstruction (F1 and F2) to confirm double label neurons.

We determined the number of BrdU/NeuN and BrdU/GFAP-IR positive cells in the anterior granular layer of the AOB. Photomicrographs were taken with a 25X objective, and a z-stack of 12 slices was obtained using a confocal microscope Zeiss LSM. Colocalization was analyzed with the orthogonal tool in the Zen 2012 software (Fig 2).

The percentage of colocalization was calculated as the number of BrdU/NeuN or BrdU/GFAP-IR positive cells multiplied by 100 and divided by the number of BrdU-IR positive cells as previously described [14, 16, 25].

### Statistical analysis

Sexual behavior parameters that were normally distributed were analyzed by a one-way ANOVA followed by Fisher post-hoc test. Those not normally distributed were analyzed by a Kruskal-Wallis test followed by a Mann-Whitney U-test. To compare data only evaluated in the pacing groups (return latencies and percentage of exits) we used a Mann-Whitney U-test. The number of cells was analyzed by a one-way ANOVA for each layer, and in case of significant effects we used a Fisher post-hoc test. For the comparison of double label cells, a t-test was done. The percentage of cells was analyzed with a chi square.

## Results

### Sexual behavior

As expected, females that did not pace the sexual interaction received a higher number of mounts [F(2,29) = 7.83; p<0.01] and intromissions [F(2,29) = 6.25; p< = 0.01] than females that paced the sexual interaction (Table 1).

**Table 1:** Sexual behavior parameters of the different groups of animals. Data represent the mean (± SEM).

Sexual Behavior Parameters	No-pacing (n = 10)	Pacing saline (n = 10)	Pacing-naloxone (n = 10)
**Mounts**	37.5 ± 7.1 *	11.7 ± 3.0	19.4 ± 2.4
**Intromissions**	35.6 ± 3.6 *	21.6 ± 1.5	16 ± 1.8
**Ejaculations**	3.1 ± 0.4	2.7 ± 0.4	1.8 ± 0.4
**Mount latency (s)**	157.9 ± 102.8	96.3 ± 23.3	215.0 ± 58.1
**Intromission latency (s)**	207.2 ± 102.0	148.5 ± 46.6	370.6 ± 127.4
**Ejaculation latency (s)**	1488.1 ± 324.1	1212.7 ± 203.8	1205 ± 187.2
**Postejaculatory interval**	448.7 ± 38	500.4 ± 50.2	575 ± 51.7
**Inter-intromission interval (s)**	67.3 ±14.4	72.5 ± 12.1	90.2 ± 21.9
**Mean lordosis intensity**	1.8 ± 0.1	1.8 ± 0.1	1.8 ± 0.1
**Lordosis quotient (%)**	99.8 ± 0.8	100.0	99.89 ± 0.2
**Mount return latency**		19.4 ± 3.9	42.1 ± 9.4 +
**Intromission return latency**		59.3± 10.2	159.7 ± 53.8 +
**Ejaculation return latency**		175.2±35.4	221.1±51.1
**% Exits after mounts**		53.4 ± 9.2	48.4 ± 8.5
**% Exits after intromissions**		75.6 ± 6.0	60.6 ± 8.6

* Different from both pacing groups, p<0.05. One-way ANOVA followed by Fisher

+ Different from pacing group, p<0.05. Mann-Whitney U test.

No differences were observed in other parameters between the females that paced the sexual interaction and those that did not. In the groups that paced the sexual interaction, the mount (U = 25.50; p = 0.034) and intromission (U = 22.00; p = 0.038) return latencies were significantly longer in the females treated with naloxone than those treated with saline before mating. The return latency was longer and the percentage of exits was higher after intromissions than after mounts. Furthermore, the ejaculation return latency was longer than the intromission return latency. These results are consistent with those previously described in the literature. Females from the 3 groups that mated were equally receptive as indicated by the lordosis quotient and mean lordosis intensity.

### BrdU immunoreactivity

**Fig 3:**
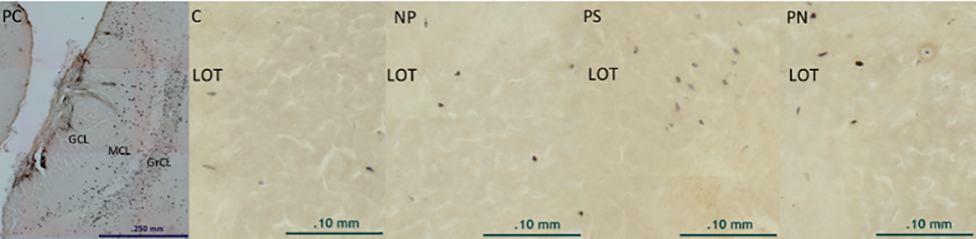
Representatives photomicrographs of BrdU-IR positive cells (dark brown dots) reveal with the diaminobenzidine in the granular cell layer of the accessory olfactory bulb in the different groups of animals. (PC) The layers of the accessory olfactory bulb (AOB) are represented in a photomicrograph of a positive control for the ICC, the animal received BrdU injections on prenatal days 15–18. A higher magnification of the GrCL is shown in the different groups: (C) Control females that did not mate. (NP) Females that mated without pacing the sexual interaction. (PS) Females that pace the sexual interaction and were injected with saline. (PN) Females that pace the sexual interaction and were injected with naloxone.Glomerular Cell Layer (GCL); Mitral Cell Layer (MCL), Granular Cell Layer (GrCL) and Lateral Olfactory Tract (LOT).

**Fig 4:**
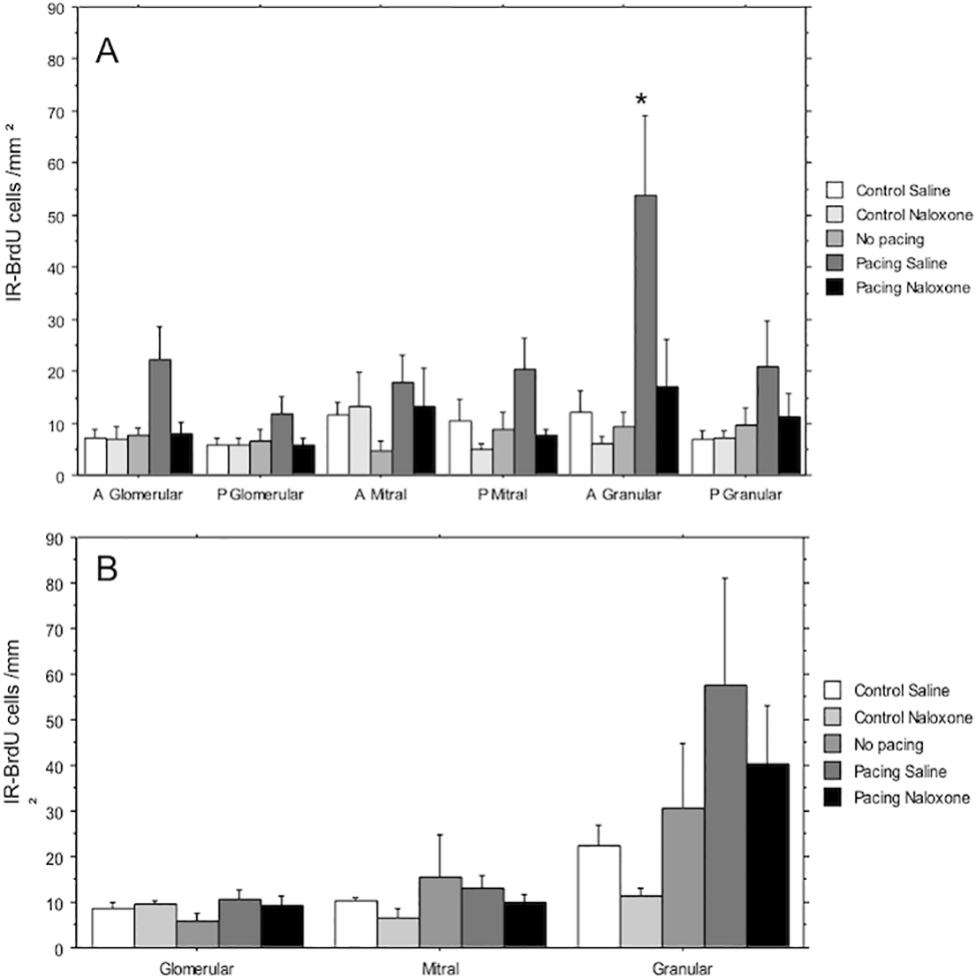
Number of BrdU-IR positive cells in the different layers of the AOB and MOB. (A) Glomerular (anterior and posterior) Mitral (anterior and posterior) and Granular (anterior and posterior) cells layers in the AOB. (B) Number of cells in the Glomerular, Mitral and Granular layers of the MOB. Two groups of animals did not mate and were injected with either saline (control saline) or naloxone (control naloxone). One group of females mated without controlling the sexual interaction (no pacing) and 2 groups controlled the rate of the sexual interaction and were injected with saline (pacing saline) or naloxone (pacing naloxone). N = 10 per group. * Significantly different from the C group in the same layer p<0.05.

A higher number of BrdU-IR cells was found in the anterior granular layer of the AOB (F 84,34) = 5.26, p = 0.002) (Figs 3 and 4A). These results confirm previous observations [15, 16]., naloxone treatment completely blocked the increase in the number of BrdU-IR cells. No differences were found in the number of BrdU-IR cells within the different layers of the MOB (Fig 4B).

### Double labeled cells

Since we only found differences in the number of BrdU-IR cells in the anterior granular layer of the AOB, we randomly selected 3 subjects of the pacing saline and pacing naloxone group to analyze with immunofluorescence the density of BrdU-IR cells and the number and percentage of BrdU/NeuN-IR cells in the anterior granular layer. As shown in Table 2, the density of BrdU-IR cells (t = 3.12; p = 0.035) and the number of BrdU/NeuN-IR cells (t = 2.87; p = 0.045) was significantly higher in the pacing saline group in comparison to the pacing naloxone females. The difference in the percentage of BrdU/NeuN-IR cells did not reach statistical significance (Chi square 3.53, p = 0.60) but the reduction in the pacing naloxone group was in the expected direction. Not enough number of new cells expressing GFAP were found to performed a statistical analyzes.

**Table 2:** Number of BrdU-IR cells and of BrdU/NeuN-IR cells as well as percentage of BrdU/NeuN-IR cells in the anterior granular layer of the AOB in females that paced the sexual interaction after an injection of saline or naloxone.

	Density of BrdU-IR cells	Density of BrdU/NeuN-IR cells	Percentage of BrdU/NeuN-IR cells.
Pacing saline (n = 3)	184.4±26.6	64.0±6.2	35%
Pacing naloxone (n = 3)	63.83±16.2 *	14.2±7.1*	22%

*Different from pacing saline group p = 0.006

## Discussion

The results of the present experiment further confirm previous observations indicating that paced mating induces a higher number of cells that differentiate into neurons in the AOB, 15 days after the paced mating encounter [15, 16]. They also confirm that if females mate without controlling the sexual interaction, no increase in the number of cells or neurons is observed. The results also demonstrate that naloxone injected prior to the paced mating test blocks the induction of new cells and neurons that reach the AOB. The control group injected with naloxone did not show a reduction in the basal number of neurons in any of the regions analyzed, suggesting that the opioid antagonist preferentially blocked the cells induced by paced mating. Together, these results suggest that opioids mediate the induction and differentiation of new cells into neurons induced by paced mating.

Several lines of evidence clearly indicate that opioids are involved in different aspects of sexual behavior ([26–28] for reviews). For example, it is generally accepted that opioid agonists inhibit male and female sexual behavior in rodents [26, 28]. Endogenous opioid peptides that bind to the μ receptor have the same inhibitory effect upon sexual behavior in males [29, 30] and females [31, 32] than synthetic opioids. There is also indirect evidence indicating that opioids are released during sexual behavior in both male and female rodents. Different groups have shown that sexual behavior in male rats [33, 34] and vaginal cervical stimulation in female rats produces analgesia [35–37] which is blocked by the administration of naloxone [33, 38]. These and other observations led to the proposal that the release of endogenous opioids during the course of sexual activity serves two functions: one, to facilitate ejaculation (in the case of the male) and two, to enhance the reinforcing properties of mating [26, 39]. In line with this proposal previous studies have shown that after rats mate opioid receptors are internalized in brain regions important in the control of sexual behavior such as the medial preoptic area, indicating ligand-induced receptor activation. The administration of NX before mating blocked the internalization of opioid receptors in rats demonstrating that opioids are released in the MPOA during sexual behavior [40]. These results, taken together with those of CPP and paced mating described in the introduction, suggest that a common opioid system mediates sexual reward [see [27] for a discussion].

As described in the introduction, the i.p. injection of NX before mating blocks the CPP induced by mating in male and female rats. This could explain the longer return latencies after mounts and intromissions observed in the group treated with NX in the present experiment. Sexual interaction is not rewarding for females and they take a longer time after sexual stimulation to return to the male side. It has also been shown that the infusion of this opioid antagonist in the MPOA blocks the rewarding stated induced by mating in males [41]. In the case of the female, we demonstrated that the infusion of NX in the MPOA, the ventromedial hypothalamus (VMH) and the medial amygdala (ME), areas important in the control of sexual behavior, blocked the reward state induced by paced mating in female rats [9]. It was suggested that opioid release in the MPOA is necessary for the positive affective state induced by paced mating and the blockade of opiate receptors in the VMH and ME interferes with the sensory input to the MPOA (see [9] for a discussion). In fact it has been suggested that the MPOA receives inputs from the VMH and ME via the bed nucleus of the stria terminalis (BNST) acting as a central node that sends outputs to neuroendocrine structures modulating behavior and prolactin secretion [42].

A third function of opioid release during sexual behavior could be to trigger the signals that will induce the formation of new cells and eventually their differentiation into neurons. This could be done by different mechanisms that need to be evaluated in future experiments. One possibility is through the connections of brain regions controlling sexual behavior with neurogenic zones of the lateral ventricles (LV) or the rostral migratory stream (RMS). To our knowledge, these connections have not been studied but there is evidence indicating that brain regions important for mating, like the MPOA, project to areas adjacent to the SVZ such as the septal region, the BNST and the caudate putamen [43, 44]. Moreover, different lines of evidence indicate that opioids can directly modulate neurogenesis in different species. Not only the administration of compounds that interact with the opioid system modulate neurogenesis (see introduction), it has been shown that μ and *k* opioid receptors play a modulatory role in the differentiation of stem-cell-derived neuronal progenitors [45] and that μ opioid receptor proteins are expressed in neuroepithelia of the lateral ventricles and radial glia in the embryonic mouse brain [46]. Mu and delta opioid receptors are expressed in the ventricular zone of adult zebra finches [47] as well, and opioid receptors and mRNA of different opioids are expressed in the proliferative zones in the fetal rat brain [48]. Based on the evidence described above, it is possible that opioids released during paced mating could directly influence proliferative zones and induce neurogenesis.

Indirect mechanisms could also be involved in the neurogenesis induced by opioids after paced mating. As already described, females that paced the sexual interaction showed higher levels of prolactin (PRL) released between 20 to 60 minutes after mating than females that did not pace the sexual interaction. It has been demonstrated that μ opioid receptors modulate PRL release [49] and those located in the MPOA play a key role [50]. It has also been shown that the administration of PRL induces neurogenesis through PRL receptors located in the SVZ [51]. There is also evidence indicating that the neurogenesis induced in female mice by male pheromones is mediated by PRL [52] and the MPOA is seen as a probable site of action [53]. Another possible indirect mechanism by which opioids could induce neurogenesis is through the interaction with neurotrophic factors. It has been shown that endogenous opioids upregulate the brain derived neurotrophic factor mRNA through the delta and μ opioid receptors [54] and the intraventricular administration of growth factors for 7 days in adult mice increased the production of new olfactory neurons [55].

To summarize, the results of the present experiment demonstrate that the neurogenesis induced by paced mating is blocked by the administration of NX before mating. The opioid antagonist itself did not modify basal neurogenesis in the olfactory bulb suggesting that the neurogenesis induced by paced mating is opioid dependent. Further experiments need to confirm this hypothesis.

## Supporting information

S1 Dataset Sexual behavior. (XLSX)

S2 Dataset ICC. (XLSX)

S1 Protocol ICC Protocol in English. (PDF)

S2 Protocol Immunofluorescence Protocol in English. (PDF)

S3 Protocol ICC Protocol in Spanish. (PDF)

S4 Protocol Immunofluorescence Protocol in Spanish. (PDF)
