# Supplementary material for: Neurogenesis in the olfactory bulb induced by paced mating in the female rat is opioid dependent
Source: PLoS One. 2017 Nov 6;12(11):e0186335. doi: 10.1371/journal.pone.0186335 (PMC5673160; doi:10.1371/journal.pone.0186335)
Supplement: S1 Protocol — (PDF) [file pone.0186335.s003.pdf]

Immunohistochemistry for BrdU

Date

1) TBS washes 5 x 3 min each

2) 30 min 1 % triton X in TBS with 1 % H202, shaker cold.

3) TBS washes 4 x 3 min each

4) 60 min 2N HCL (RT)

5) TBS washes 2 x 3 min each

6) 15 min 0.5% sodium borohydride (1g/200 ml TBS)

7) TBS washes 4 x 3 min each

8) 30 min in TBS albumin (10%) and Tx (0.3%)

9) Primary Antibody BrdU. Anti mouse 1:2,000 in TBS albumin (1 %) and TX (0.32%) on shaker cold, minimum 16 hrs.

10) TBS washes 4 x 3 min each with TX (0.02%) and albumin (1%)

11) Biotinylated 2ndary antibody 1:500 in TBS, albumin (1%) and TX (0.32%) 2 hrs shaker, RT

12) TBS washes 4 x 3 min each with TX (0.02%)

***Half an hour before made up elite ABC (2 drops of A, 2 drops of B in 10 ml)

13) Sections in ABC 90 min, shaker RT

14) TBS only washes 4 x 3 min each

15) DAB as indicated in the kit or according to the solution below

16) TBS washes 3 x 3 min each

DAB solution for 50 ml, 45 ml of deionized water, 30 mg of DAB 36 microliters of H_2O_2 (30%) add 5 milliliters of nickel solution (1%).
