# Supplementary material for: Neurogenesis in the olfactory bulb induced by paced mating in the female rat is opioid dependent
Source: PLoS One. 2017 Nov 6;12(11):e0186335. doi: 10.1371/journal.pone.0186335 (PMC5673160; doi:10.1371/journal.pone.0186335)
Supplement: S2 Protocol — (PDF) [file pone.0186335.s004.pdf]

Immunohistochemistry for BrdU/NEUN/GFAP fluorescence

Date

DAY 1

1) PBS washes 4 x 10 min each

2) 15 min 0.5% sodium borohydride in PBS

3) PBS washes 3 x 10 min each

4) 30 min 1 % triton X in PBS with 1 % H202

5) 10 min in DMSO (1%) in PBS

6) PBS washes 3 x 10 min each

7) 60 min 2N HCL (RT)

8) PBS washes 3 x 10 min each

9) 15 min 0.5% sodium borohydride in PBS

10) PBS washes 3 x 10 min each

11) 30 min in PBS albumin (10%) and Tx (0.3%)

12) Primary Antibody BrdU. Anti rat 1:800 (serotec) in PBS albumin (1 %) and TX (0.32%) minimum 20 hrs.

DAY 2

13) PBS washes 3 x 15 min each with TX (0.02%) and albumin (1%)

14) Biotinylated 2ndary antibody, anti rat IgG 1:500 in PBS, albumin (1%) and TX (0.32%) 3 hs

15) PBS washes 3 x 10 min each with TX (0.02%)

***Half an hour before made up elite AB (2 drops of A, 2 drops of B in 10 ml PBS)

16) Sections in AB 90 min,

17) PBS washes 3 x 10 min each

18) kit cumarina 1:100 **slides in the dark**

19) PBS washes 2 x 10 min each

20) Primary Antibody NeuN mouse 1:250 in PBS albumin (1 %) and TX (0.32%)
minimum 20 hrs.

DAY 3

21) PBS washes 3 x 15 min each with TX (0.02%) and albumin (1%)

22) Biotinylated 2ndary antibody, anti mouse IgG 1:300 in PBS, albumin (1%) and TX (0.32%)
3 hs

23) PBS washes 3 x 10 min each with TX (0.02%)

***Half an hour before made up elite AB (2 drops of A, 2 drops of B in 10 ml PBS)

24) Sections in AB 90 min,

25) PBS washes 3 x 10 min each

26) kit CY3 1:100 **slides in the dark**

27) PBS washes 2 x 10 min each

28) Primary Antibody GFAP rabbit 1:500 in PBS albumin (1 %) and TX (0.32%)
minimum 20 hrs.

DAY 4

29) PBS washes 3 x 15 min each with TX (0.02%) and albumin (1%)

30) 2ndary antibody, anti rabbit Alexa 1:250 green in PBS, albumin (1%) and TX (0.32%)
3 hs

31) PBS washes 3 x 10 min each with TX (0.02%)

32) PBS washes 2 x 10 min each
