# Supplementary material for: Neurogenesis in the olfactory bulb induced by paced mating in the female rat is opioid dependent
Source: PLoS One. 2017 Nov 6;12(11):e0186335. doi: 10.1371/journal.pone.0186335 (PMC5673160; doi:10.1371/journal.pone.0186335)
Supplement: S3 Protocol — (PDF) [file pone.0186335.s005.pdf]

Inmunohistoquímica para BrdU especie rata

Fecha:
Grupo:

1. Lavados con TBS (3 min c/u)					
2. Incubarcon TBS, Tritón X (1%) y H_2O_2 (1%) (30 min)					
3. Lavar TBS (3min)					
4.Incubar con 2N HCL 37°C (1h)					
5.- Lavar con TBS (3 min)					
6. Incubación con TBS y borohidrato de sodio (0.5%) (15 min)					
7. Lavados con TBS (3 min c/u)					
8. Incubadocon TBS, albumina (10%) y TX (0.3%) (30 min)					
9. ANTICUERPO PRIMARIO<br>Anti ratón 1:2,000<br>en TBS, albumina (1%) y TX (0.32%) (16 hrs mínimo)					
10. Lavados TBS con TX (0.02%) y albumina (1%) (5 min c/u)					
10. ANTICUERPO SECUNDARIO<br>Anti.IgG mouse: 1:500					
en TBS, albumina (1%) y TX (0.32%) (2 hrs)					
11. Lavados con TBS y TX (0.02%) (5 min c/u) preparar ABC					
	preparar ABC				
12. Incubar ABC (2 gotas de A, 2 gotas de B en 10ml) (90 min)<br>*preparar con media hora antes de usarse. Nota guardar un poco para verificar que la reacción funciona					
13. Lavados con TBS (3min c/u)					
14. Revelado con DAB (10 min máx)					
15. Lavados con TBS (3 min c/u)					

Solucion de DAB para 50ml
45mil de agua desionizada
30mg de DAB
36microlitros de H202 al 30%
solucion refrigerar al utilizar agregar
5mil de solucion de nikel al 1%

Nike 1gr en 100ml de agua tridestilada o desionizada.
