# Supplementary material for: Neurogenesis in the olfactory bulb induced by paced mating in the female rat is opioid dependent
Source: PLoS One. 2017 Nov 6;12(11):e0186335. doi: 10.1371/journal.pone.0186335 (PMC5673160; doi:10.1371/journal.pone.0186335)
Supplement: S4 Protocol — (PDF) [file pone.0186335.s006.pdf]

Inmuno amplificación BrdU/NEUN/GFAP fluorescencia

1er DÍA
1. Lavados con PBS (10 min c/u)
2. Incubar con PBS y borohidrato de sodio (0.5%) (15 min)
3. Lavados con PBS (10 min c/u)
4. Incubar con PBS, Tritón X (1%) y H2O2 (1%) (30 min)
5. Incubar con PBS y DMSO (1%) (10 min)
6. Lavados con PBS (10 min c/u)
7. Incubar con 2N HCL (60 min) 37°C
8. Lavado con PBS (10 min)
9. Incubar con PBS y borohidrato de sodio (0.5%) (15 min)
10. Lavados con PBS (10 min c/u)
11. Incubar con PBS, Albúmina 10% y TX (0.3%) (30 min)
**12. ANTICUERPO PRIMARIO** **Anti-BrdU (rata) serotec 1:800** PBS, Albúmina 1% y TX (0.32%) (20 hrs mínimo) a 4ºC,.
**2do DÍA**
13. Lavados PBS con TX (0.02%) y Albúmina 1% (15 min)
**14. ANTICUERPO SECUNDARIO** Anti rata IgG biotinilado _______________ vector 1:500 en PBS, Albúmina 1% y Tx (0.32%) (3 hrs)
15. Lavados con PBS y TX (0.02%) (10 min c/u) PREPARAR COMPLEJO AB
16. Para 10mil de PBS (2A 2B)- TX 0.32% incubar por 90 min
17. Lavados con PBS (10 min c/u)
18. Kit Cumarina 1:100 (portaobjetos **Sin Gelatinizar**) A partir de aquí OSCURAS!!! CÁMARA HÚMEDA
19. Lavar con PBS (10 min)
20. Incubar anticuerpo primario* en PBS-TX 0.32%, albúmina 1% (20 hrs a 4ºC) **NeuN ratón (1:250)**

Inmuno amplificación BrdU/NEUN/GFAP fluorescencia-2

3er DÍA			
21. Lavar con PBS-TX 0.02%, albúmina 1% (15 min)			
22**. ANTICUERPO SECUNDARIO** Anti mouse IgG biotinilado _____________ vector 1:300 en PBS, Albúmina 1% y Tx (0.32%) (3 hrs)			
23. Lavados con PBS y TX (0.02%) (10 min c/u) **PREPARAR** COMPLEJO **AB**			
24. Para 10mil de PBS (2A 2B)- TX 0.32% incubar por 90 min			
25. Lavados con PBS (10 min c/u)			
26. Kit CY3 1:100 (portaobjetos **Sin Gelatinizar**) A partir de aquí OSCURAS!!! CÁMARA HÚMEDA			
27. Lavar con PBS (10 min)			
28. Incubar anticuerpo primario* en PBS-TX 0.32%, albúmina 1% (20 hrs a 4ºC) GFAP conejo **(1:500)**			
**4to DÍA**			
29. Lavar con PBS-TX 0.02%, albúmina 1% (15 min)			
30. Anticuerpo secundario ** en PBS-TX 0.32%, Albúmina 1% (3hrs). Alexa 488 antirabbit (1:1250 VERDE)			
31. Lavar con PBX-TX 0.02% (10 min)			
32. Lavar con PBS (10 min)			
